# Investigation of the Mechanical Properties of Quick-Strength Geopolymer Material Considering Preheated-to-Room Temperature Ratio of Sand, Na_2_SiO_3_-to-NaOH Ratio, and Fly Ash-to-GGBS Ratio

**DOI:** 10.3390/polym15051084

**Published:** 2023-02-21

**Authors:** Mohammad Rizwan Bhina, Kuang-Yen Liu, John-Eric Hsin-Yu Hu, Chih-Ta Tsai

**Affiliations:** 1 Department of Civil Engineering, National Cheng Kung University, Tainan 70101, Taiwan; 2 Sustainable Environment Research Laboratories (SERL), National Cheng Kung University, No. 500, Sec. 3, An-Ming Road, Annan District, Tainan 709015, Taiwan

**Keywords:** preheated sand, geopolymer material, quick strength, compressive strength, Na_2_SiO_3_-to-NaOH ratio, fly ash-to-slag ratio, thermal resistance

## Abstract

Geopolymer concrete is a useful alternative construction material for bridge deck systems, as it is characterized by a low carbon footprint, rapid setting, quick strength development, low cost, freeze-thaw resistance, low shrinkage, and sulphate and corrosion resistance. Heat curing enhances the mechanical properties of geopolymer materials (GPM), but it is not suitable for large structures, as it affects construction activities and increases energy consumption. Therefore, this study investigated the effect of preheated sand at varying temperatures on GPM compressive strength (Cs), the influence of Na_2_SiO_3_ (sodium silicate)-to-NaOH (sodium hydroxide—10 molar concentration), and fly ash-to-granulated blast furnace slag (GGBS) ratios on the workability, setting time, and mechanical strength properties of high-performance GPM. The results indicate that a mix design with preheated sand improved the Cs of the GPM compared to sand at room temperature (25 ± 2 °C). This was caused by the heat energy increasing the kinetics of the polymerization reaction under similar curing conditions and with a similar curing period and fly ash-to-GGBS quantity. Additionally, 110 °C was shown to be the optimal preheated sand temperature in terms of enhancing the Cs of the GPM. A Cs of 52.56 MPa was achieved after three hours of hot oven curing at a constant temperature of 50 °C. GGBS in the geopolymer paste increased the mechanical and microstructure properties of the GPM as a result of different formations of crystalline calcium silicate (C-S-H) gel. The synthesis of C-S-H and amorphous gel in the Na_2_SiO_3_ (SS) and NaOH (SH) solution increased the Cs of the GPM. We conclude that a Na_2_SiO_3_-to-NaOH ratio (SS-to-SH) of 5% was optimal in terms of enhancing the Cs of the GPM for sand preheated at 110 °C. Additionally, as the quantity of ground GGBS in the geopolymer paste increased, the thermal resistance of the GPM was significantly reduced.

## 1. Introduction

Approximately 1 kg of cement production releases around 0.8 Kg of CO_2_ into the atmosphere and requires 7500 megajoules (MJ) of energy. Furthermore, the demand for concrete is increasing by approximately 3% each year due to booming construction and development in the road, bridge, and infrastructure industries. This makes concrete the second most used material worldwide after water [1,2]. Hence, various researchers have investigated whether geopolymer concrete has the potential to fully or partially replace cement. It offers energy-effective and rapid solutions for the construction industries, for example, for replacing and repairing bridge deck expansion joints and road pavements. It has three main advantages: (1) it reduces CO_2_ emissions and energy demands in the cement industries; (2) it is a form of industrial waste utilization; (3) it produces quick-setting, high-strength repair materials [3,4,5].

Davidovits [6] developed a new cementitious material known as geopolymer concrete. The strength of this product results from the polymerization process. In GPM, low-calcium FA (FA) and ground granulated blast-furnace slag (GGBS) are used as a binder, and a strong base such as NaOH/KOH is used as an activator at room temperature. NaOH/KOH is known as an alkaline activator. The bonding property in GPM results from the development of an amino-silica gel produced by a chain reaction, which is related to the leaching process between the binder (FA) and activator (NaOH/KOH). In the leaching process, NaOH/KOH leaches out Al^+3^ and Si^+4^ ions from FA when mixed with FA and produces aluminosilicate gel with good binding properties. Therefore, the concentration and type of alkali activator and the curing temperature play essential roles in the polymerization reaction, controlling the mechanical properties, durability, workability, flow rate, and geopolymer concrete’s initial and final setting time.

Additionally, failure and degradation of bridge deck expansion joints are serious problems for bridge engineers. Expansion joint repair and restoration work often requires divers or restricting the traffic flow, which causes obvious inconveniences. Moreover, the maintenance and repair of bridge decks, pavements, and highways are costly. High maintenance costs increase because the material used in repair work is costly as a result of its high-strength and quick-setting properties. Therefore, a sustainable and economical repair material to quickly restore bridge deck expansion joints is of great importance [7,8].

In the case of quick-strength geopolymer concrete, the rapid setting is a significant issue [9]. Wang [10] observed that geopolymer concrete based on alkali-activated slag (AAS) starts setting within 15 min, and that quick setting significantly affects the workability. Previous studies indicated that hot curing enhanced the mechanical properties of geopolymer material. Various researchers revealed that the hot curing process could produce high-strength concrete materials if applied in the early stages. However, the heat curing method for GPM may not be suitable for large structures as it can affect construction activities, and long periods of hot curing increase energy consumption and, thus, the cost of the structure. Won et al. [11] produced a rapid-setting material with a Cs of approximately 22 MPa in 4 h. The Cs of geopolymer concrete can be enhanced by increasing the curing time and temperature. However, high-temperature curing of full-scale structures is challenging, and extended hot curing increases the energy consumption and cost of the structure [12,13]. Hardjito et al. [14,15] concluded that geopolymer materials cannot possess high-strength and quick-setting characteristics when produced quickly under room-temperature curing conditions.

The hot mixing process of the GPM can enhance the polymerization reaction rate in the initial stage [16]. Ground granulated blast-furnace slag also plays a vital role in developing rapid-setting, and quick-strength geopolymer material as a higher slag content improves the Cs and reduces the setting time [17]. Therefore, the current study investigated the effect of preheated sand at varying temperatures by utilizing the GGBS in developing a quick-strength geopolymer material. The optimum preheated temperature for developing a geopolymer material with high Cs was also investigated.

Geopolymer mortar (GPM) requires different mass proportions of FA, GGBS, SS (sodium water glass), and SH (sodium hydroxide) solutions to produce high Cs [18]. Many studies on slag-based geopolymer concrete state that quick setting and poor workability are the biggest problems in applying GGBS-based GPM in the construction of large structures [9]. Several researchers also include sodium silicate (Na_2_SiO_3_ (water glass)) in the inorganic geopolymer concrete as an activator because it is also an alkaline solution. Increasing the quantity of SS may improve the mechanical strength, setting time, and workability of the GPM. The primary purpose of adding sodium silicate is to provide sufficient silica to form the Si-O-Si linkage, thus improving the Cs. The silica modulus of SiO_2_: Na_2_O is the main characteristic of Na_2_SiO_3_ (SS). However, understanding the exact mechanism of water glass when used as a multifunctional agent (providing an alkaline environment and a source of key material) in GPM remains unknown, and the relationship between the Cs, flow rate, and setting time needs to be further explored. In addition, no specific guidelines or methods have yet been developed, and researchers are using alternative methods for determining the values of high-performance inorganic geopolymer concretes [19].

Furthermore, the SS-to-SH ratio influences the GPM setting time, workability, and Cs [19,20]. The SS-to-SH ratios of 1, 2.5, and 0.4 produced geopolymer concretes of 70 MPa, 56.8 MPa, and 17.3 MPa, respectively [9,20]. However, the effect of the Na_2_SiO_3_/NaOH ratio on the workability, setting time, and Cs with preheated fine aggregate remains to be elucidated. The main contribution of this study is to explore how various SS-to-SH geopolymer mortar ratios affect the setting time, workability, flow rate, and compressive strength of the GPM. The present study also investigated the optimum Na_2_SiO_3_-to-NaOH ratio to produce rapid Cs with preheated sand and hot curing at 50 °C. The Cs of fresh geopolymer mortar were investigated at 1 h (1 h), 2 h (2 h), 3 h (3 h), and 1 d (1 d). In the present study, we used NaOH at 10 molarities and sodium water glass (nSiO_2_Na_2_O) with a modulus of 2.88 as an activator to calculate the optimum ratio of Na_2_SiO_3_-to-NaOH. It was shown that the Cs of concrete also depended on porosity properties. The Cs decreased with the increase in porosity and water absorption. SEM and EDS were used for the micro- and macrostructure analyses of the GPM.

The aim of the current research was to investigate the effect of preheated sand on the compressive strength (Cs) of the GPM in order to overcome the problem of hot curing applied to a large structure. Moreover, the goal of this study was to develop an early-high-strength GPM and analyze its properties, taking into account the effects of the preheated fine aggregate, compressive strength, different fly ash-to-GGBS ratios, and different Na_2_SiO_3_-to-NaOH ratios. Furthermore, the thermal resistance of the GPM was also investigated in this research. However, modeling and experimental investigations of joint expansion failure were not taken into account in the current study.

## 2. Materials and Methods

**Figure 1:**
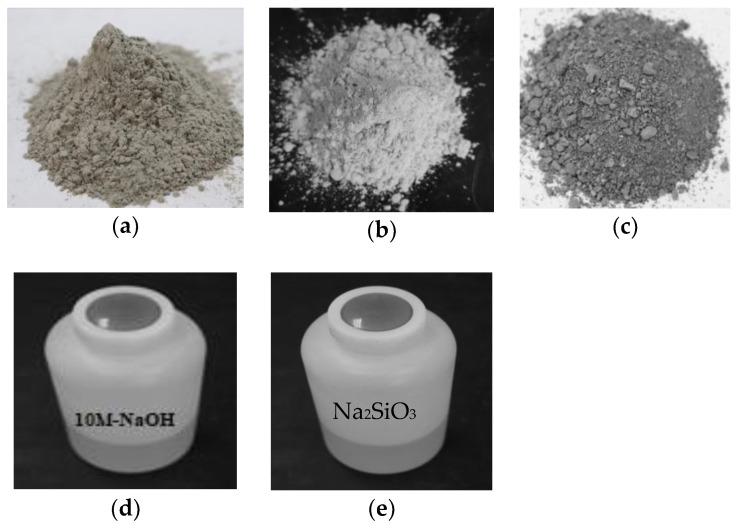
The main constituents of the geopolymer mixture: are (**a**) fly ash (FA); (**b**) GGBS; (**c**) river sand; (**d**) 10 M-NaOH; (**e**) Na_2_SiO_3_.

**Figure 2:**
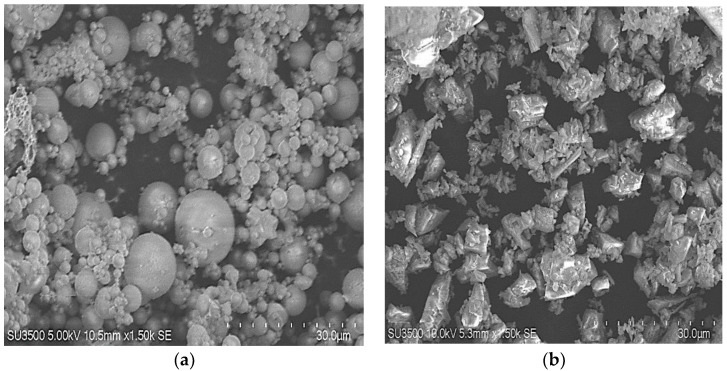
SEM results: (**a**) class-F fly ash (FA); (**b**) S-4000 GGBS.

The main constituent used to develop the geopolymer mixture shown in Figure 1 was industrial waste, which is readily available in Taiwan. Low-calcium fly ash (Class-F) [21] and S4000 GGBS were used as a binder (silica and alumina material), and sodium hydroxide (alkali solution) with a molarity of 10 (SiO_2_/Na_2_O = 1.28) was used as an activator to develop the geopolymer paste. The fundamental morphological analysis of fly ash (FA) and GGBS materials was performed using energy-dispersive X-ray spectroscopy (EDX) and scanning electron microscopy (SEM). The results of the SEM and EDX analyses of FA and GGBS are shown in Figure 2. Viscous and transparent liquid sodium silicate, with 28–30 w% of SiO_2_ and 9–10 w% of Na_2_O with a specific gravity of 1.526 g/cm^3^ and pH value of 12, was purchased from RongXiang industrial limited, Taiwan. Table 1 shows the specification of the commercial-grade liquid sodium silicate (Na_2_SiO_3_). The energy dispersive X-ray spectroscopy (EDX) analysis indicated that the percentages of SiO_2_, Al_2_O_3,_ and CaO (by mass %) in the FA and GGBS were 58.0, 25.47, and 0.0; and 21.64, 8.53, and 68.11, respectively (see Table 2). It was observed that NaOH produced better results than KOH due to its high solubility in geopolymer synthesis reactions [22]. We used sodium hydroxide (NaOH) in liquid form with a 97% purity as an alkali activator in this study, manufactured and supplied by Sun Tech industries, Taiwan.

**Figure 3:**
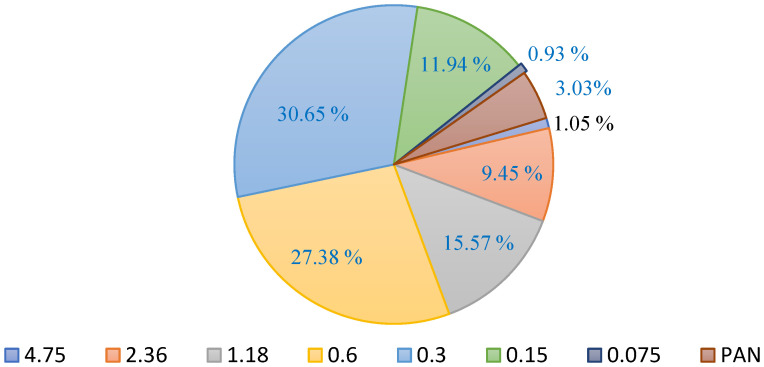
Percentage of natural river sand retained in individual sieves.

**Figure 4:**
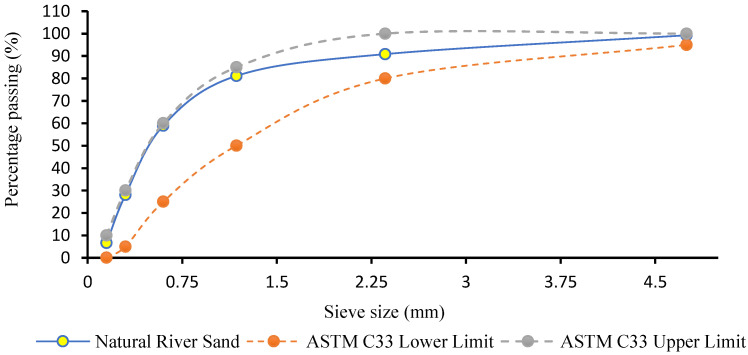
Sieve analysis of natural river sand.

**Figure 5:**
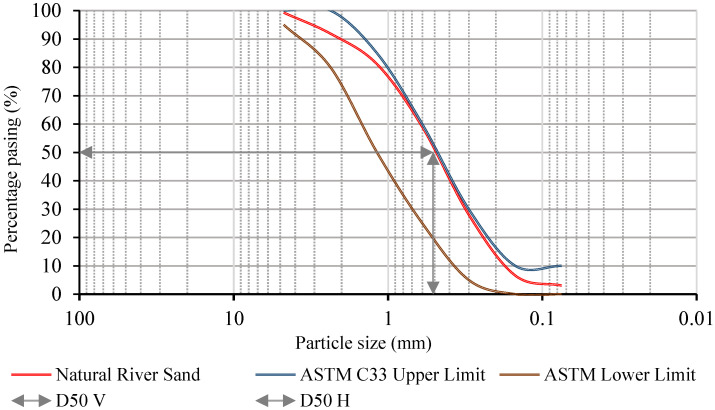
Natural river sand particle size distribution.

As a result of the aberration force on the surface of roads and pavements, ASTM C33/C33M 2011a indicates that particle sizes finer than 75 μm should not make up more than 5% of the mass of the fine aggregate used for the repair material [23]. Figure 3 shows the percentage of natural river sand particles retained in individual sieves. It also shows that the mass percentage of particles finer than 75 μm was less than 3%. Figure 4 and Figure 5 show the natural river sand’s particle size distribution as per ASTM C33/C33M 2013a [23]. The result of the sieve analysis revealed that the grading of sand is under the upper and lower limit, as indicated by ASTM C33/C33M 2013a [23]. The results of the phase 1 Cs test for the GPM showed that fine aggregate preheated to 110 °C was the optimal heating temperature in terms of enhancing mechanical strength. Natural river sand preheated at 110 °C for 24 h with a maximum particle size of 4.75 mm was used to prepare the GPM paste for the phase 2 test. The aggregate was entirely absorbent, and all moisture was eliminated by curing it overnight (about 12 to 16 h) at a temperature of 110 ± 5 °C (ASTM D2216-19) [16,24]. Table 3 shows the physical characteristics of FA, GGBS, and natural river sand. The specific gravities, water absorption, and fineness modulus of the main constituents of GPM have been calculated as per ASTM standards (see Table 3). The FA, GGBS, and sand exhibited specific gravities of 1.45, 2.59, 2.63 (saturated surface-dry), and 2.76 (oven dry), respectively. The water absorption and fineness modulus (FM) of the fine aggregate was calculated to be 2.88 (%) and 2.63, respectively.

### 2.1. Mix Proportions

**Figure 6:**
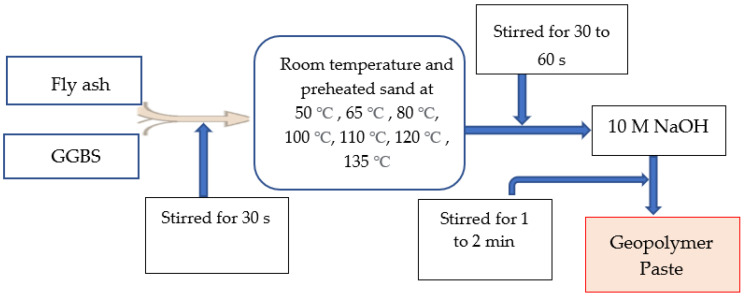
The experimental concept of the phase 1 geopolymer paste.

**Figure 7:**
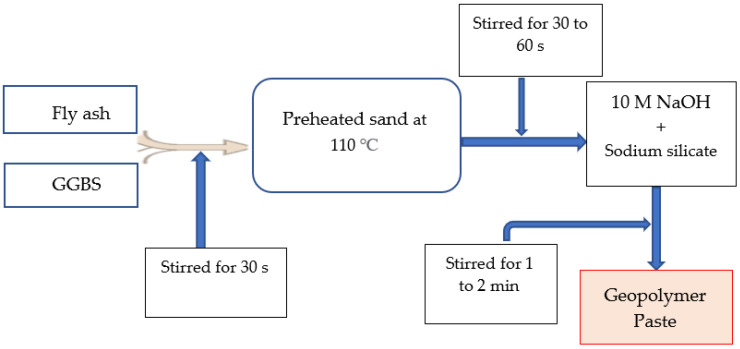
The experimental concept of the phase 2 geopolymer paste.

The geopolymer material paste was prepared in two phases by mixing FA and GGBS as a binder with 10 M NaOH (SiO_2_/Na_2_O 1.28) as an activator and preheated sand as a fine aggregate. The mixing process of the main constituents of the geopolymer paste is shown in Figure 6 and Figure 7. Figure 6 indicated the phase 1 mis design and Figure 7 represented phase 2 mix design of GPM paste. Firstly, in phase 1 (see Figure 6), FA and GGBS were mixed and stirred for 30 s. Then, the preheated sand (heated at 50 °C, 65 °C, 80 °C, 100 °C, 110 °C, 120 °C, and 135 °C for 6 h) was mixed into the FA and GGBS powder and stirred for 30 to 45 s to remove the lumps in the sand. Thereafter, 10 M NaOH was mixed into the FA, GGBS, and sand mixture and stirred for 1 to 3 min to ensure its consistency. The specimens ID 10M-GPM-0, 10M-GPM-50, 10M-GPM-65, 10M-GPM-80, 10M-GPM-100, 10M-GPM-110, 10M-GPM-120, and 10M-GPM-135 were designated phase 1 samples. In the second phase (see Figure 7), FA and GGBS were mixed and stirred for 1 to 2 min. Then, the sand was heated at 110 °C for 6 h, mixed into the FA and GGBS powder, and stirred for 30 to 45 s to remove the lumps. Thereafter, 10 M NaOH and No.3 sodium silicate with 28 w% of SiO_2_ and 9 w% of Na_2_O with a modulus of 2.88 were mixed into the FA, GGBS, and sand mixture and stirred for 1 to 3 min to ensure consistency. The consistency of the prepared geopolymer paste was observed when mixing the SH and SS into the FA, GGBS, and preheated sand mixture. The specimens ID GPM-3H-A1 to GPM-3H-C5 were designated phase 2 samples. All mechanical properties of the GPM were investigated after hot oven curing at 50 °C as per the curing period. Additionally, before testing, all specimens were maintained at room temperature (28 ± 2 °C) for 30 min to minimize thermal stress.

It was noted that the presence of preheated sand enhanced the geopolymer material’s rapid-setting properties. Additionally, the quantity and ratio of FA to GGBS played a vital role in the strength generation of the geopolymer material [25]. We used 10 M SH (NaOH) in the current research as it was noted in the literature review that 10 M and 12 M NaOH, used as an activator, produced better mechanical strength results in terms of workability and durability [22]. A detailed explanation of the fly ash-to-GGBS, alkali activator-to-binder, solid-to-liquid, and the binder-to-sand ratio is given in Table 4, showing the mass ratios for all the geopolymer material mix designs. As can be seen, the SS-to-SH ratio and the FA-to-GGBS ratio (by mass) changed from 0.0% to 30% and 1:1 to 1:3 in the phase 2 mix design.

### 2.2. Casting of Specimen and Curing

High-temperature curing and preheated sand were adopted in this research, as elevated curing accelerates the polymerization reaction rate, which may enhance the mechanical strength of the GPM [26]. A machine-driven mixture with a rotational speed of 285 ± 10 rpm was used to prepare the geopolymer material (GPM) paste. The GPM Cs test was performed on a cube of 5 × 5 × 5 cm as per ASTM C109/109M-16 [27]. GPM specimens were cast by pouring GPM paste into a steel 5 × 5 × 5 cm mold before being compacted using a wooden strip and a table vibrator. The geopolymer material specimen was later maintained in a hot air oven for 1 h, 2 h, 3 h, and 1 d at 50 °C. Before testing, all samples were de-molded and stored at room temperature for 15 min.

### 2.3. Test Procedure

Geopolymer materials have recently been adopted as repair materials in the bridge industry. A GPM can only be used as a repair material if it exhibits quick strength development, rapid setting quality, acceptable workability and flow rate, and sufficient slant shear strength. In this research, we performed compressive strength, flow rate, and apparent porosity tests in phase 1 in order to evaluate the effects of preheated sand at various temperatures on the Cs of the GPM. The Cs, flow rate, setup time, and thermal stability characteristics of the GPM material with different SS-to-SH ratios (%) and FA-to-GGBS ratios were examined in this study. The Cs tests were performed on 5 × 5 × 5 cm cube specimens after 1 h, 2 h, 3 h, and 1 d as per ASTM C109/109M-16 [28]. A universal testing machine UH-C 100 A (Shimadzu) based on ASTM C109/109M-16 in the structural laboratory of the civil engineering department, National Cheng Kung University, was used for the Cs test with a constant loading rate of 2.5 kgf/cm^2^/s. The average result from three specimens is reported as the Cs of each mix. Each specimen was maintained in a hot air oven for 1 h, 2 h, 3 h, and 1 d at a constant temperature of 50 °C.

The X-ray diffraction (XRD), energy dispersive X-ray spectroscopy (EDS), and scanning electron microscopy (SEM) analyses were performed to evaluate the microstructure properties of the GPM. Moreover, the influence of the SS-to-SH and FA-to-GGBS ratios on the microstructure properties of the GPM was observed. The samples for the XRD, EDS, and SEM analyses were collected in the form of GPM powder after the compressive strength test using the GPM cube specimens at 1 D. Furthermore, the GPM paste powder was crushed in a pulverizer and then passed through a 75 mm sieve. Then, it was maintained in a sealable container (desiccator). XRD, EDS, and SEM analyses were performed at the core facility center, the micro-/nanotechnology division, National Cheng Kung University, Taiwan. The XRD patterns of the GPM fine powder sample were produced at 2θ = 5–90°, using a step size of 0.03° and a scan speed of 0.5 s/step. At 1 day, fine powder specimens from the GPM paste were subjected to an energy dispersive spectroscopy (EDS) analysis. In order to identify the types of elements contained in samples on the microscale, e.g., 1 mm, SEM and EDS analyses were performed. To examine the variation in the gel composition in the GPM under normal conditions with various SS-to-SH and FA-to-GBS ratios, GPM element compositions were collected from four locations in the paste areas.

## 3. Results and Discussion

### 3.1. The Impact of Preheated Sand

**Figure 8:**
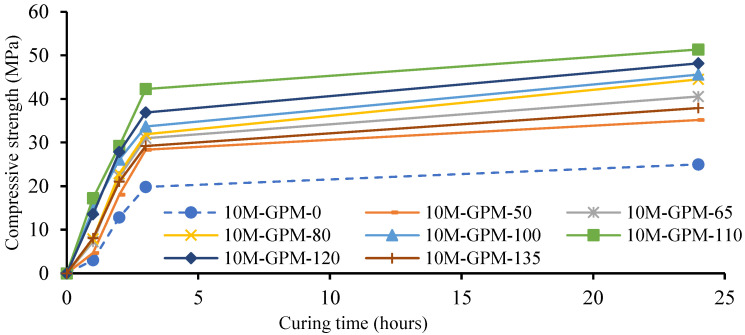
Compressive strength test results of the geopolymer material (GPM) using room-temperature sand and preheated sand after 1 h, 2 h, 3 h, and 1 d of hot air curing at a constant temperature of 50 °C.

Experimental tests of the phase 1 geopolymer paste were performed to calculate the effect of preheated sand at various temperatures on the Cs of the geopolymer material (GPM). Figure 8 shows the results of the Cs test of the geopolymer material (GPM) using room-temperature sand and sand preheated at 50 °C, 65 °C, 80 °C, 100 °C, 110 °C, 120 °C, and 135 °C as a fine aggregate. The Cs of the GPM was calculated after 1 h, 2 h, 3 h, and 1 d of hot air curing at a constant temperature of 50 °C. As can be observed from Figure 8 and Table 5, the specimen cast with preheated sand exhibited a better performance as compared to the specimen produced with room-temperature sand in terms of Cs at 1 h, 2 h, 3 h, and 1 d. The Cs and growth rate in the case of the preheated sand may have been the result of the heat energy provided by the preheated sand. The heat energy enhanced the polymerization reaction rate by triggering the diffusion of molecules and increasing the rate of the molecule’s conversion into monomers. The heat energy also works as a catalyst that increases the rates of chemical reactions, which creates large amounts of polymer. These polymers connect in a comprehensive 3D chain system, improving the GPM’s compressive strength. This may have been partially due to the reaction of GGBS with the alkaline solution being an exothermic reaction, i.e., that generates heat, and this heat energy promotes the polymerization reaction. Therefore, the GPM paste developed with preheated sand exhibited a high early strength as compared to the GPM paste produced with room-temperature sand.

**Figure 9:**
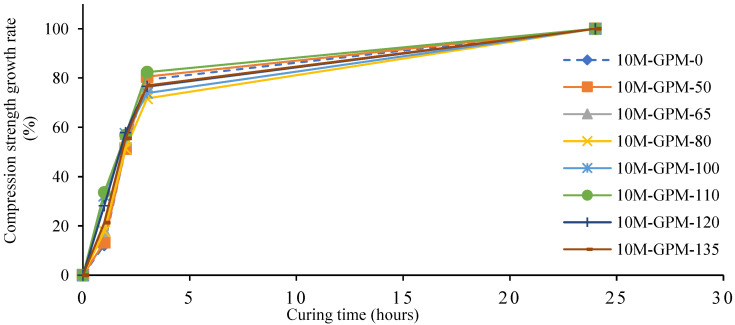
Compressive strength growth rate (%) of the geopolymer material (GPM) using room-temperature sand and preheated sand after 1 h, 2 h, 3 h, and 1 d of hot air curing at a constant temperature of 50 °C.

Furthermore, as can be seen in Figure 9, the GPM specimens cast with preheated sand at 110 °C yielded higher Cs of 17.23 MPa, 29.19 MPa, 42.31 MPa, and 51.36 MPa at 1 h, 2 h, 3 h, and 1 d, respectively. This is due to GGBS and the rate of water evaporation. GGBS plays a governing role in developing geopolymer materials with quick strength development and rapid setting characteristics [17]. Increasing the quantity of GGBS enhanced the Cs of the GPM and reduced the setting time and flow rate [17,29]. Heat energy increases the reaction kinetics of the geopolymer material but also intensifies the rate of water evaporation in the geopolymer paste, which leads to autogenous shrinkage and produces micro capillaries in the GPM [30]. In addition, water evaporation also reduces the quantity of water in the hot GPM paste. In this scenario, the calcium oxide (CaO) present in the GGBS is required water to form the C-S-H and C-A-H gel quickly [31].

Additionally, Figure 9 shows the Cs growth rate of the geopolymer material using room-temperature sand and preheated sand after 1 h, 2 h, 3 h, and 1 d of hot air curing at a constant temperature of 50 °C. Figure 9 reveals that the Cs growth rate of all GPM specimens continues to increase with time. However, preheated sand at 110 °C significantly affected the GPM’s Cs growth rate. This may have been due to the sand preheated at 110 °C not being as large. This may have resulted in the water from the hot GPM mix not evaporating quickly and the formation of the C-S-H gel rate increasing, as was observed in the optimum system. Therefore, Figure 8 and Figure 9 suggest that 110°C was the optimal temperature for preheated sand to enhance GPM’s compressive strength.

**Figure 10:**
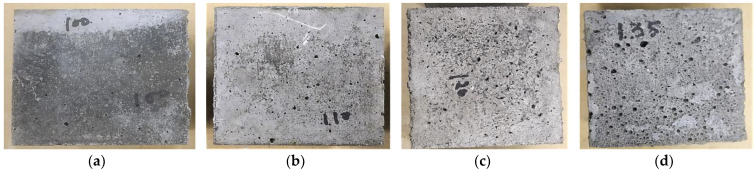
Geopolymer material specimens using preheated sand: (**a**) preheated sand at 100 °C; (**b**) preheated sand at 110 °C; (**c**) preheated sand at 120 °C; (**d**) preheated sand at 135 °C.

**Figure 11:**
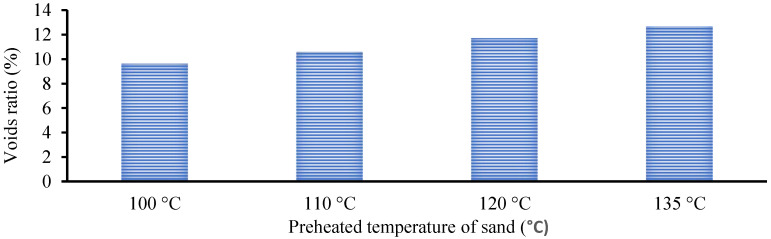
Voids ratio (%) of the geopolymer material specimens using preheated sand at 100 °C, 110 °C, 120 °C, and 135 °C.

Figure 10 shows that raising the preheated sand’s temperature increased the voids ratio in the GPM specimens, decreasing the geopolymer material’s bulk density. This may have been due to water evaporation, as the water evaporation rate accelerates as the temperature increases, which significantly enhances the voids and capillaries in the GPM [32]. The porosity (%) of the GPM prepared using preheated sand was calculated using a 5 × 5 × 5 cm cube specimen. The porosity (%) was calculated using Equation (1) as per ASTM C1688/C1688M-14a [33], and the average results of three specimens were recorded. The pore distribution analysis showed that the void ratio (%) increased as the preheat temperature of the sand increased, which affected the GPM’s mechanical characteristics. An increase in the preheat temperature increased the void in the geopolymer material (see Figure 11).

(1) $$V\;\left(\%\right)=\frac{V_{p}}{V_{b}}\times 100$$

where V is the void ratio (%), V_p_ is the pore volume of specimens, and V_b_ is the bulk volume of specimens.

### 3.2. Impact of the SS-to-SH (%) Ratio on the Mechanical Strength of the GPM with Sand Preheated at 110 °C

**Figure 12:**
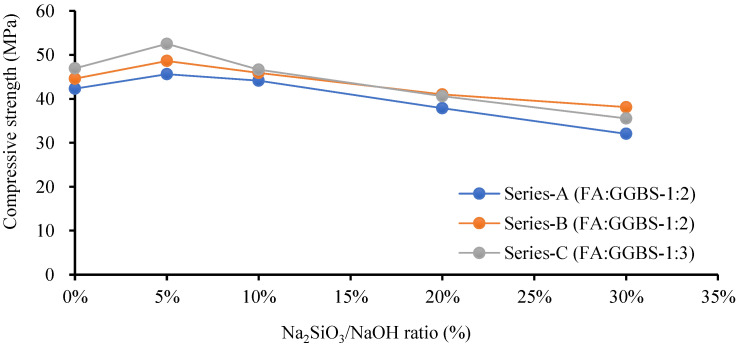
Compressive strength test results for the geopolymer specimen using various Na_2_SiO_3_-to-NaOH and FA-to-GGBS ratios with sand preheated at 110 °C and cured in a hot oven at a uniform temperature of 50 °C for 3 h.

Several studies illustrated that GPM paste produced a lower mechanical strength at an ambient temperature using NaOH (SH) or Na_2_SIO_3_ (SS) as an alkaline activator. The Cs of the GPM may be increased using a blend of SH and SS as an activator with a mixture of blended FA and GGBS as a binder [34]. GPM’s quick strength development depends on the binder and activator type [35]. The SS-to-SH (%) ratio may be increased to enhance the mechanical qualities of the GPM, which enhances the dissolution rate of CaO, silica, and alumina present in the GGBS and FA. The dissolution growth rate accelerates the polymerization rate to enhance the GPM’s Cs. Experimental tests using the phase 2 geopolymer paste were performed to calculate the effect of the SS-to-SH (%) ratio with sand preheated at 110 °C (see Figure 7). The current study also investigated the impact of various FA-to-GGBS ratios on the Cs of the geopolymer material (GPM) with various Na_2_SiO_3_-to-NaOH ratios. All GPM specimens were hot cured in the oven at a temperature of 110 °C for 3 h. Figure 12 shows the influence of the SS-to-SH and FA-to-GGBS ratios on the Cs of the GPM with sand preheated at 110 °C after curing in a hot oven at a constant temperature of 50 °C for 3 h. This indicated that 5% SS-to-SH ratio was the most optimized ratio to enhanced the Cs of GPM.

**Figure 13:**
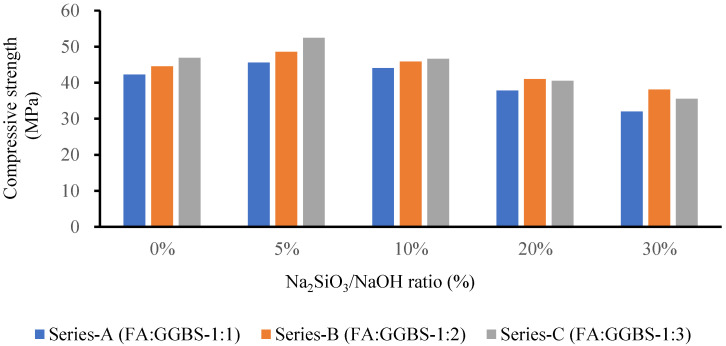
Compressive strength test results for the geopolymer specimen using various Na_2_SiO_3_-to-NaOH and FA-to-GGBS ratios with sand preheated at 110 °C and cured in a hot oven at a uniform temperature of 50 °C for 3 h.

Figure 13 shows that increasing the amount of GGBS enhanced the rapid Cs development of the GPM. The GPMs with FA-to-GGBS ratios of 1:1, 1:2, and 1:3 resulted in Cs of 42.31 MPa, 44.59 MPa, and 46.95 MPa, respectively, in 3 h. The Cs of the GPM was increased by the increase in the amount of GGBS in the GPM mix design [17]. This may have been due to the large amount of GGBS added to the additional CaO, SiO_2,_ and Al_2_O_3_ in an amorphous state in the GPM paste, producing a considerable amelioration in the Cs [34,36]. Additionally, GGBS, a glassy phase, can react faster than FA to form the C-S-H gel. This may have been partially because the interaction between the GGBS and the alkaline solution was an exothermic reaction, i.e., it produced heat, and this heat energy aided in the polymerization process. Increasing the GGBS concentration enhanced the GPM paste’s Cs [18].

**Figure 14:**
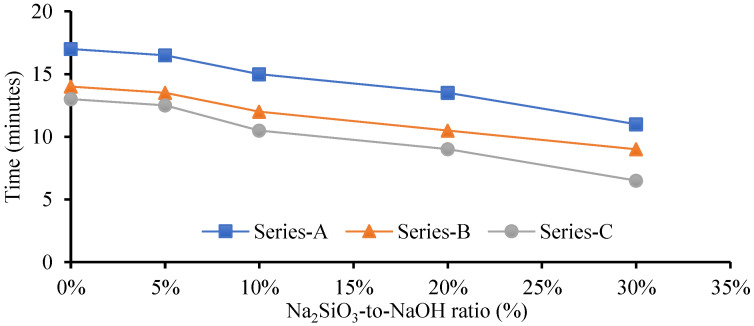
Geopolymer material initial setting time using various Na_2_SiO_3-_to-NaOH and FA-to-GGBS ratios with sand preheated at 110 °C.

**Figure 15:**
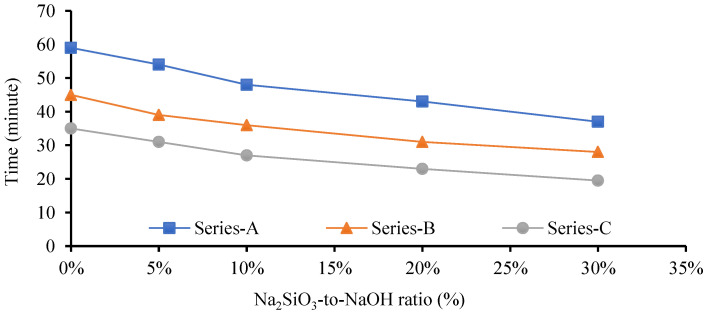
The final setting time of the geopolymer material using various Na_2_SiO_3_-to-NaOH and FA-to-GGBS ratios with sand preheated at 110 °C.

Several researchers found that FA and GGBS mixed as a binder and an SS and SH mixture as an activator produced higher mechanical GPM strength. Using a NaOH and Na_2_SiO_3_ (sodium silicate) combination enhanced the silica content in the GPM paste, which accelerated the polymerization reaction. The highest Cs of 52.52 MPa was achieved with Na_2_SiO_3_-to-NaOH and FA-to-GGBS ratios of 5% and 1:3, respectively, with sand preheated at 110 °C and cured in a hot oven at a constant temperature of 50 °C for 3 h. The Na_2_SiO_3_-to-NaOH and FA-to-GGBS ratio of 10% and 1:3 produced a Cs of 46.67 MPa, which was 11.13% lower than that of the SS-to-SH ratio of 5% with the same amount of FA, GGBS, and the same hot curing period. This may have been because adding an extra amount of sodium silicate (Na_2_SiO_3_) decreased the GPM compressive strength, the setting time (see Figure 14 and Figure 15), the workability, and the flow rate (see Table 6). Standard deviation and coefficient of variation are the important values for sample variability considered for the current study. Due to the fact that it takes into consideration both the mean and the standard deviation, the coefficient of variation is seen to be a more useful measure of variability. The study’s results show exceptionally low values for the standard deviation and coefficient of variance. In this investigation, the range of the GPC coefficient of variation varied from 0.59% to 10.92%. For a common test like the compressive strength of concrete, the value of CoV is under the acceptable level (see Table 6) [37]. The amount of SS (Na_2_SiO_3_) must be substantial and optimized in the GPM paste [38]. A large SS-to-SH ratio led to decreased GPM Cs. The surplus formation of OH^-^ in the GPM paste may be responsible for the deterioration in the Cs [39]. Additionally, the preheated temperature of the sand enhanced the hardening rate of Na_2_SiO_3_ due to the heat energy [40,41], and sodium silicate increased water molecule loss, which generated an excessive amount of C-A-S-H and N-A-S-H gel. Therefore, in the presence of preheated sand, an upsurge in the Na_2_SiO_3_ content reduced the workability and flow rate of the GPM paste, decreasing the Cs. It was also observed that the alkali activator Na_2_SiO_3_-to-NaOH ratio of 5% was the most effective ratio with which to improve the Cs of the GPM paste with sand preheated at 110 °C and cured in a hot oven at a uniform temperature of 50 °C for 3 h.

### 3.3. Effect of the Molar Ratio (SiO_2_/Al_2_O_3_)

Nath et al. [42] revealed that the molar ratio also influenced the mechanical properties of the geopolymer material (GPM). Table 6 shows the deviation in the Cs of the GPM associated with the molar ratio (SiO_2_/Al_2_O_3_) with sand preheated at 110 °C and cured in a hot oven at 50 °C. Assuming that the molar ratio (SiO_2_/Al_2_O_3_) was a governing factor in enhancing the Cs of the GPM, as can be seen in Table 6, the most favorable molar ratios (SiO_2_/Al_2_O_3_) were 2.46 and 2.49, which produced Cs of 48.61 MPa and 52.52 MPa in 3 h, respectively, with sand preheated at 110 °C and cured in a hot oven at 50 °C. Several researchers investigated the molar ratio (SiO_2_/Al_2_O_3_) and concluded that a molar ratio (SiO_2_/Al_2_O_3_) of up to 2.87 was most effective in improving the Cs of the GPM [39]. Nevertheless, adding a high amount of GGBS decreases the Cs of the GPM [43]. A previous study suggested that increasing the amount of Na_2_SiO_3_ in the GPM improved its mechanical strength due to the increase in the SiO_2_/Al_2_O_3_ ratio [44]. However, in the case of preheated sand, increasing the ratio of SiO_2_/Al_2_O_3_ (see Table 6) adversely affected the strength gain of the GPM because, in the presence of preheated sand, the crystallization rate increased, which negatively affected the workability and flow rate.

### 3.4. Microstructure Study of the Phase 2 GPM

The relationship between the microstructure and Cs development in the GPM at various Na_2_SiO_3_-to-NaOH (SS-to-SH) and FA-to-GGBS ratios with sand preheated at 110 °C and cured in a hot oven at a uniform temperature of 50 °C for 3 h was assessed.

#### 3.4.1. EDS Analysis

**Figure 16:**
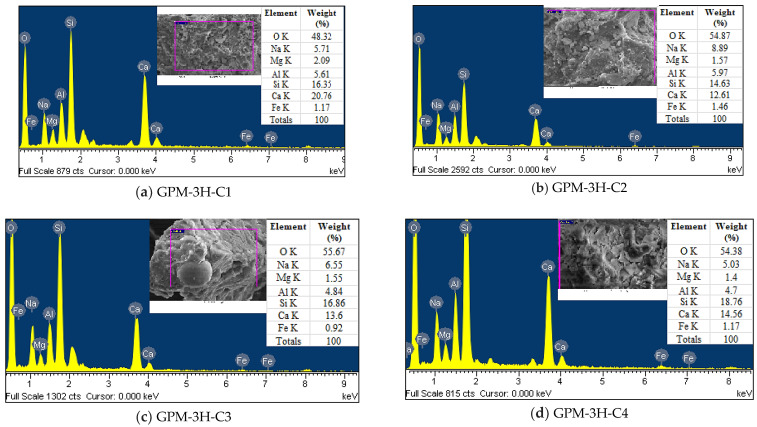

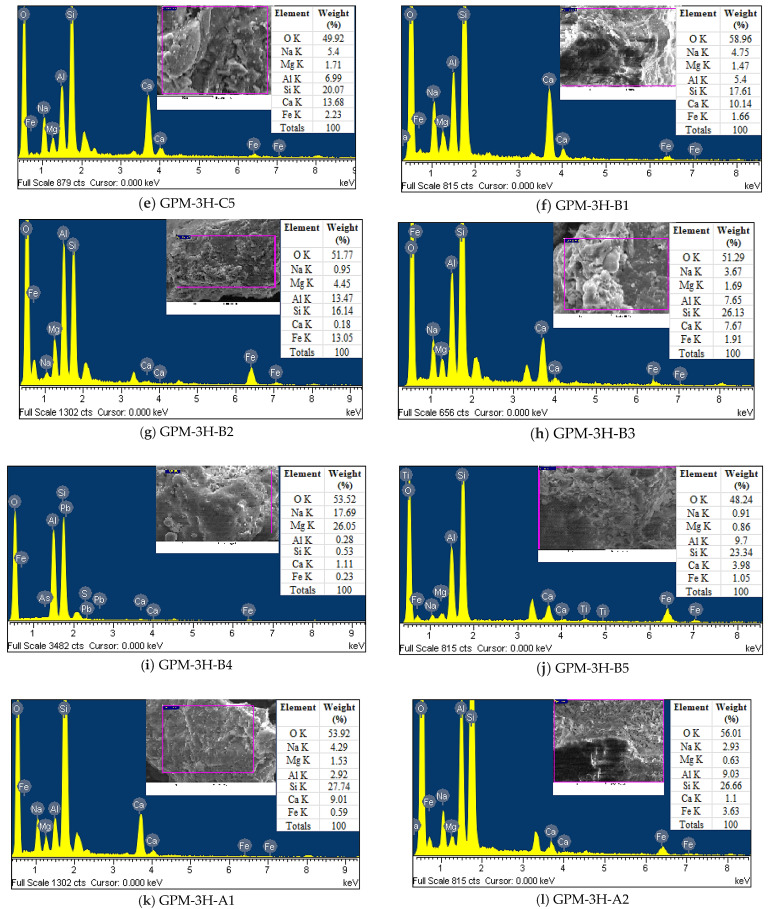

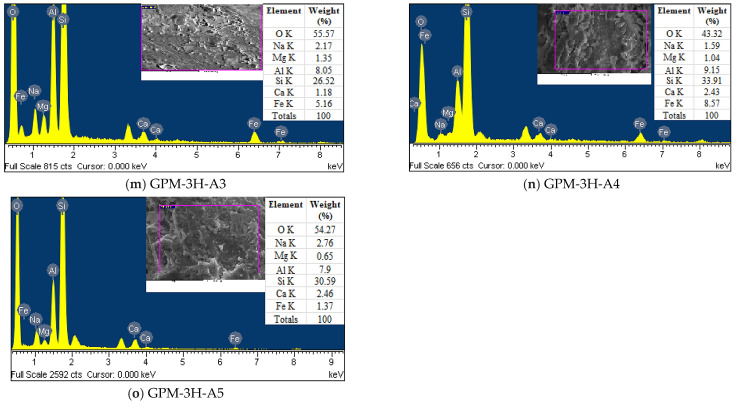
EDS spectra of the GPM paste using various Na_2_SiO_3_-to-NaOH and FA-to-GGBS ratios with sand preheated at 110 °C and cured in a hot oven at a uniform temperature of 50 °C for 3 h and 1 d of room-temperature curing: (**a**) GPM-3H-C1; (**b**) GPM-3H-C2; (**c**) GPM-3H-C3; (**d**) GPM-3H-C4; (**e**) GPM-3H-C5; (**f**) GPM-3H-B1; (**g**) GPM-3H-B2; (**h**) GPM-3H-B3; (**i**) GPM-3H-B4; (**j**) GPM-3H-B5; (**k**) GPM-3H-A1; (**l**) GPM-3H-A2; (**m**) GPM-3H-A3; (**n**) GPM-3H-A4; (**o**) GPM-3H-A5.

**Figure 17:**
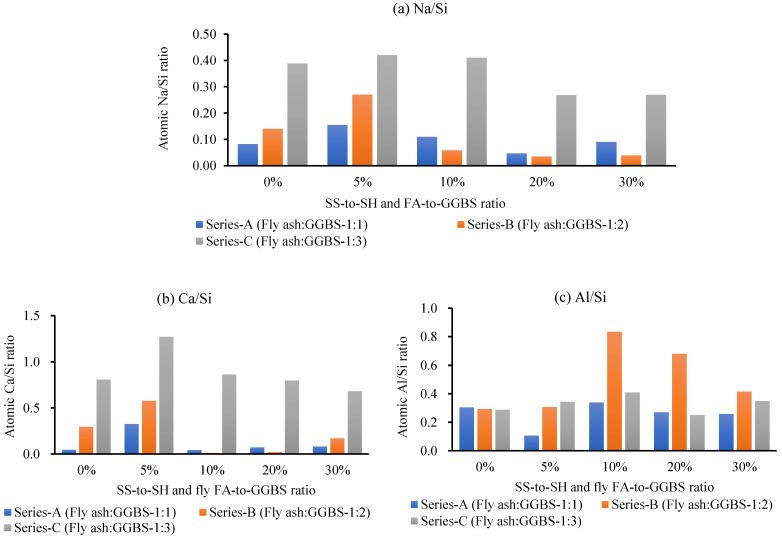
Elemental ratios obtained from the EDS analysis of the GPM mixes: (**a**) atomic Na/Si ratio; (**b**) Ca/Si ratio; (**c**) Al/Si ratio using various Na_2_SiO_3_-to-NaOH and FA-to-GGBS ratios with sand preheated at 110 °C and cured in a hot oven at a constant temperature of 50 °C for 3 h and 1 d room-temperature curing.

Figure 16 shows the EDS spectra of the GPM using various SS-to-SH and FA-to-GGBS ratios (phase 2; see Table 4). Before the SEM and EDS examinations, all specimens were cured for 1 day at room temperature after being heated for 3 h in a hot oven at a temperature of 50 °C. The EDS spectra of the GPM revealed that the major elements were oxygen, silica, alumina, sodium, and calcium. The element ratios of atomic sodium/silica, alumina/silica, and calcium/silica are shown in Figure 17. Figure 17 illustrates that the GPM mix atomic ratios of sodium/silica, alumina/silica, and calcium/silica were in the range of 0.02–0.61, 0.11–0.68, and 0.01–1.27, respectively. It was also observed that the element ratios were mostly asymmetrical with various SS-to-SH and FA-to-GGBS ratios. Thus, the GPM’s SS-to-SH and FA-to-GGBS ratios govern the variation in the atomic sodium/silica and calcium/silica ratios [45]. The GPM Series C mix design (FA-to-GGBS-1:3) generated higher Na/Si and calcium/silica ratios, i.e., (0.27–0.61) and (0.68–1.27), as compared to the GPM Series B mix design (fly ash-to-GGBS—1:2) (0.04–0.27) and (0.01–0.58), and Series A (fly ash-to-GGBS—1:1) (0.05–0.15) and (0.04–0.32), respectively. Moreover, no significant relationship or consistent variation was detected in the atomic Al/Si element ratios among the GPM Series A, Series B, and Series C mix designs. This showed that adding more GGBS to the GPM mix design, increasing the amount of CaO in the GPM paste, and introducing FA into the GPM paste helped to hasten the development of the C-H-S gel and the N-A-S-H gel in the geopolymer material.

The atomic Al/Si and Ca/Si ratios were significantly increased by creating the C-S-H and N-A-S-H gel, which improved the Cs of the GPM [45,46]. The quick strength development characteristics of the GPM resulted from the sodium alumino-silicate hydrate (N-A-S-H) gel [47]. Additionally, when examining how the SS-to-SH ratio (per cent) affected the Cs of the GPM, it was discovered that the atomic ratios of sodium-to-silica and calcium-to-silica were primarily higher for the GPM made with a 5% SS-to-SH ratio than for those made with a 10%, 20%, or 30% SS-to-SH ratio (See Figure 17a,b). The atomic Al/Si ratio exhibited unsystematic changes in the GPM (See Figure 17c). The Ca/Si and Na/Si atomic ratios for 0%, 5%, 10%, 20%, and 30% SS-to-SH ratios for Series A, Series B, and Series C were in the range of (0.15–0.05) and (0.32–0.08), (0.27–0.040) and (0.58–0.17), and (0.35–0.51) and (1.27–0.68), respectively. Therefore, all phase 2 GPM mix designs (Series A, Series B, Series C, (See Table 4)) with a 5% SS-to-SH ratio yielded higher Cs after being cured in a hot oven at a constant temperature of 50 °C for 3 h (Figure 12).

#### 3.4.2. SEM Analysis

**Figure 18:**
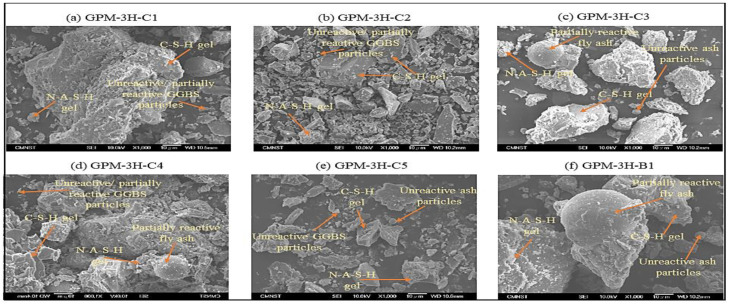

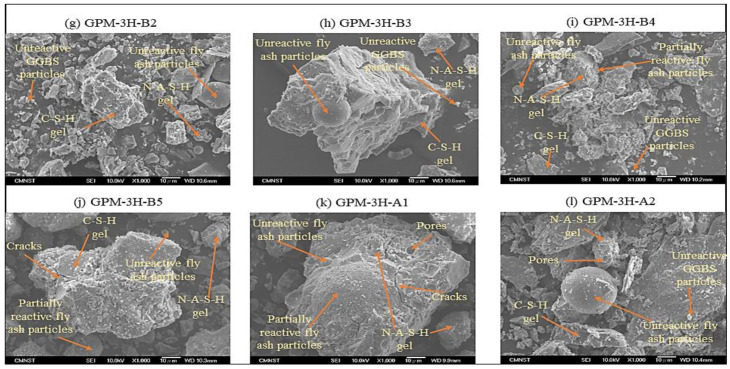
SEM picture of the GPM paste cured in a hot oven at a fixed temperature of 50 °C for 3 h while being exposed to various SS-to-SH and FA-to-GGBS ratios and 1 d room-temperature curing: (**a**) GPM-3H-C1; (**b**) GPM-3H-C2; (**c**) GPM-3H-C3; (**d**) GPM-3H-C4; (**e**) GPM-3H-C5; (**f**) GPM-3H-B1; (**g**) GPM-3H-B2; (**h**) GPM-3H-B3; (**i**) GPM-3H-B4; (**j**) GPM-3H-B5; (**k**) GPM-3H-A1; (**l**) GPM-3H-A2.

Several researchers claimed that blending FA and GGBS as a binder and SS and SH as an activator was the most effective mix design to produce geopolymer materials with a high Cs [34,48]. Additionally, FA reacts very slowly in ambient temperature curing, and hot curing is required to improve the polymerization reaction rate. The exothermic interaction between GGBS and alkaline solution, which provides heat energy and stimulates the production of the C-A-S-H and C-S-H gel, can improve the rapid setting and quick strength development characteristics in a GPM [48,49]. The SEM analysis of the geopolymer material using various SS-to-SH (%) and FA-to-GGBS ratios with sand preheated at 110 °C and cured in a hot oven at 50 °C for 3 h and 1 d room-temperature curing is presented in Figure 18. Figure 18 shows that the FA-to-GGBS ratio of 1:3 produced higher Cs with Na_2_SiO_3_ + NaOH as an activator. The Cs of 52.52 MPa was achieved with FA-to-GGBS and Na_2_SiO_3_-to-NaOH ratios of 1:3 and 5%, respectively. An FA-to-GGBS ratio of 1:3 with Na_2_SiO_3_ + NaOH produced a dense structure. The unreacted FA and GGBS particles in the GPM showed that the aluminosilicate material’s dissolution in the presence of the alkaline solution was insufficient, and the precursor materials’ chemical reaction was poorly realized [45,50]. Unreacted FA and GGBS particles, cracks, and pores were observed in GPM paste Series A (k–o) at 1 d (Figure 18). These unreactive FA and GGBS particles, pores, and cracks significantly reduced the Cs of the GPM. Unreactive or partially reactive FA-generated cavities and pores. These cavities and pores made the GPM more porous and led to the deterioration in the Cs (see Figure 18k,l). Hence, GPM Series A (k–o) achieved lower Cs as compared to Series B (f–j), and Series C (a–e) (see Figure 13). Furthermore, Figure 18 shows that Series C (a–e) and Series B (f–j) developed better homogeneous and denser microstructures as compared to Series A (k–o). It was also observed that the GPM paste with a large amount of slag produced a more homogeneous, denser, and more compact microstructure [51]. The dense, more compacted microstructure and the high Cs characteristics of the GPM may have resulted from the formation of the C-S-H gel (calcium-rich geopolymer gel) due to the high amount of GGBS used to prepare the GPM paste (see Table 4).

#### 3.4.3. XRD Analysis

**Figure 19:**
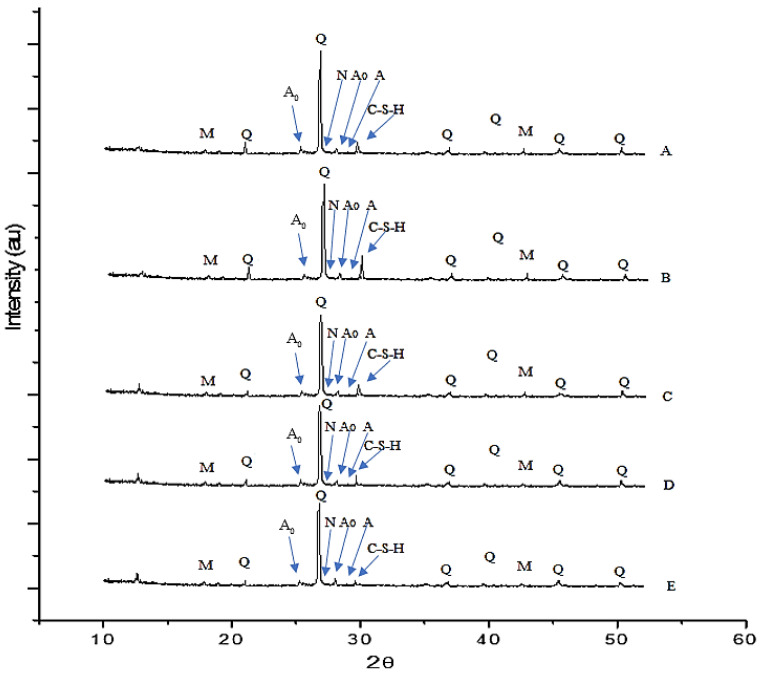
XRD analysis of the geopolymer material of Series C mix design using various SS-to-SH (%) ratios from 0 to 30% and an FA-to-GGBS ratio of 1:3 with sand preheated at 110 °C and cured in a hot oven at a constant temperature of 50 °C for 3 h and 1 d room-temperature curing. A: SS/SH-0%; B: SS/SH-5%; C: SS/SH-10%; D: SS/SH-20%; E: SS/SH-30%. SS: sodium silicate solution (Na_2_SiO_3_); SH: sodium hydroxide (NaOH); Q: quartz; M: mullite; A: albite; N: nepheline; A_0_: anorthoclase.

The XRD analysis of the Series C geopolymer material with SS-to-SH ratios of 0% to 30% and an FA-to-GGBS ratio of 1:3 (Series C mix design; see Table 4) after 3 h hot oven curing at 50 °C and 1 d room-temperature curing is presented in Figure 19. Figure 19 shows that the crystalline peaks of quartz (SiO_2_) and mullite (AL_4_.75Si_1_._25_O_9_._63_) were observed around 25° to 30° in all geopolymer material specimens [52,53]. These humps in the GPM XRD analysis confirmed the C-S-H and N-A-S-H phase development [54,55]. The crystalline peak of quartz and mullite also demonstrated the presence of unreacted or slightly reacted binder in the geopolymer specimens. In addition, the breakdown of aluminosilicate in the alkaline liquid revealed the emergence of amorphous to semi-crystalline phases. All GPM specimens subjected to an alkaline liquid-to-binder ratio exhibited peaks of albite, anorthoclase, and nepheline in the XRD analysis: the albite peaks at 22.3° 2θ and 28.2° 2θ; the anorthoclase peaks at 23.9° 2θ, 27.3° 2θ, and 35.1° 2θ; and the nepheline peaks at 26.9° 2θ. All GPM XRD results also showed a weak peak for C-S-H and calcite at 29.1° 2θ.

The authors suggest that increasing the SS-to-SH ratio (%) in the GPM mix design may reduce the geopolymer material’s Cs. The results of the XRD investigation also correlate with the GPM's Cs test (see Figure 8) using various SS-to-SH ratios with sand preheated at 110 °C and cured in a hot oven at a constant temperature of 50 °C for 3 h and 1 d room-temperature curing. The test results showed that the GPM mix design with a higher SS-to-SH ratio (%) resulted in lower compressive strength. Moreover, the development of calcium silicate hydrate and alkali aluminosilicate gels, which are responsible for improving the Cs of the GPM, was increased in the GPM mix design with a lower SS-to-SH ratio (%), as the peaks for the C-S-H gel, albite, and nepheline were observed to be higher with the lower SS-to-SH ratio (%).

### 3.5. Thermal Stability of the GPM Using Various Na_2_SiO_3_-to-NaOH and Fly Ash (FA)-to-GGBS Ratios

**Figure 20:**
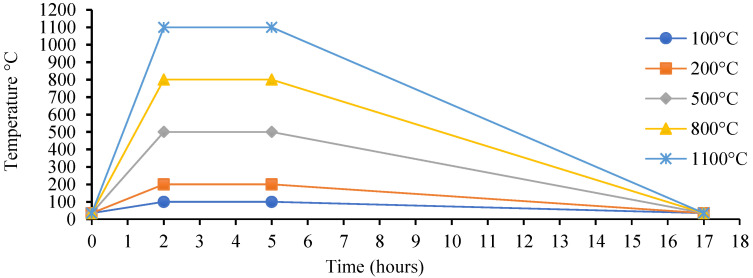
Thermal loading (°C) for the geopolymer material paste using various SS-to-SH (%) and FA-to-GGBS ratios with sand preheated at 110 °C.

The type of binder and activator used in the GPM paste and their ratios are crucial factors in establishing the characteristics of geopolymer materials [56]. This study explored the effects of the SS-to-SH ratio (%) and the FA-to-GGBS ratio on the thermal stability of the GPM. Cube specimens with an aspect ratio of 1:1 were cast to investigate the geopolymer material’s thermal stability at various temperatures. Electric furnaces were used to expose the GPM specimens to elevated temperatures (°C). Phase 2 Series A, Series B, and Series C GPM paste (see Table 4) were cured in a hot oven at a temperature of 50 °C for 3 h and then subjected to 28 days of ambient curing before being exposed to an elevated temperature to calculate the temperature stability, as per ASTM E119-18c [57]. The GPM specimens were maintained 100 °C, 200 °C, 500 °C, 800 °C, and 1100 °C for 3 h to observe the thermal stability. After being removed from the furnace, all GPM specimens were allowed to cool for 24 h at room temperature. The temperature loading of the GPM specimens is shown in Figure 20. However, an electric furnace may not produce a natural fire-loading effect. This is especially true in relation to temperature shocks [58,59].

**Figure 21:**
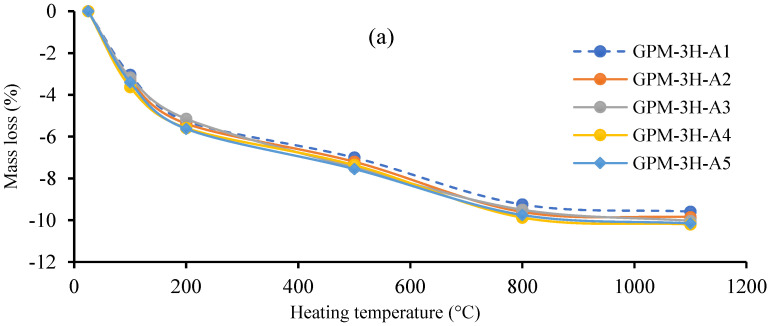

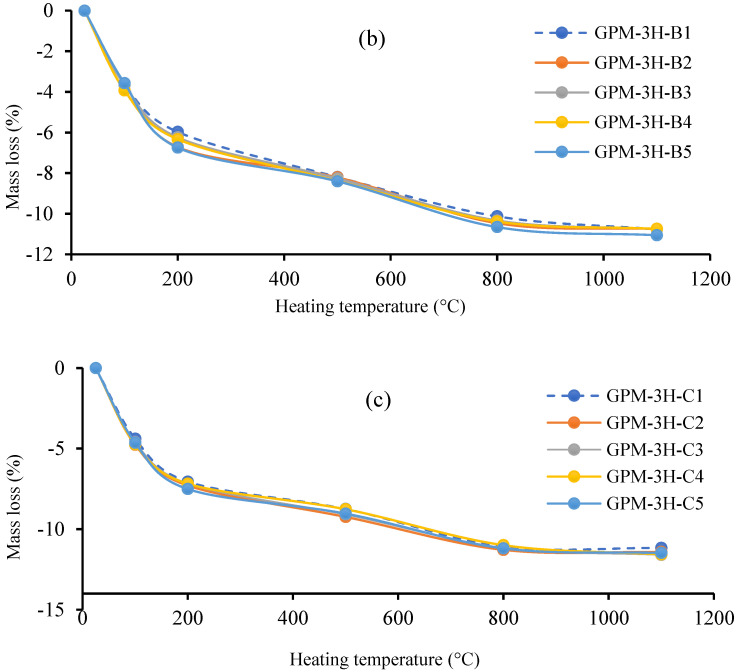
Mass loss (%) vs. elevated temperature for the geopolymer paste using various Na_2_SiO_3_-to-NaOH and FA-to-GGBS ratios with sand preheated at 110 °C: (**a**) Series A geopolymer paste; (b) Series B geopolymer paste; (**c**) Series C geopolymer paste.

Figure 21a–c shows the mass loss (%) of the Series A, Series B, and Series C GPM paste, respectively, at different temperatures. A sharp decline in the mass loss (%) was observed at 100 °C and 200 °C. This may have been due to the free water in the GPM evaporating, which occurred when the heat energy broke the binding, holding the water molecules together at 100 °C and 200 °C [59]. The mass loss rate in the GPM slowed after 200 °C, possibly because of the dihydroxylation of water molecules, which were chemically bound in the GPM paste. Figure 21 shows that increasing the percentage of GGBS in the GPM paste enhanced the thermal effect on the GPM and increased the mass loss. Additionally, GGBS is responsible for the mass formation of calcium silicate hydrates (or C-S-H) in the GPM paste, improving the rapid-strength characteristics of the GPM [17]. Hence, the decomposition of the C-S-H gel at elevated temperatures is responsible for the loss of chemically bound water molecules from the C-S-H gel, which caused a mass loss in the GPM paste [60,61,62]. Therefore, C-S-H material formation significantly enhanced the Cs of the GPM, but this may damage the GPM at elevated temperatures.

The SS-to-SH ratio (%) did not exhibit a significant effect on the thermal stability of the GPM paste. However, it can be seen in Figure 21a–c that the GPM pastes with a 0% SS-to-SH ratio (%) exhibited slightly better performance than GPM pastes with water glass and NaOH as an activator in each Series (Series A, Series B, Series C).

**Figure 22:**
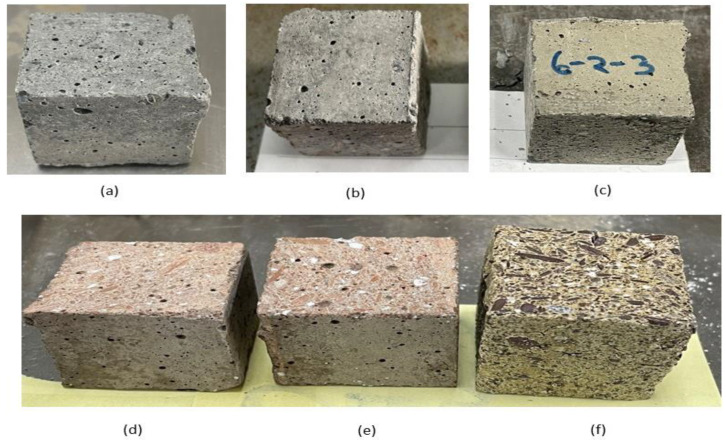
Geopolymer specimens after exposure to various heating temperatures (°C): (**a**) unexposed; (**b**) 100 °C; (**c**) 200 °C; (**d**) 500 °C; (**e**) 800 °C; (**f**) 1100 °C.

Figure 22 shows the GPM specimens after being exposed to various heating temperatures (°C): (a) room temperature; (b) 100 °C; (c) 200 °C; (d) 500 °C; (e) 800 °C; (f) 1100 °C. There was no notable change at the surface of the GPM specimens after being exposed to 100 °C. The GPM specimen surface exhibited a small amount of yellow color after heating at 200 °C, and the surface turned light red when exposed to 500 °C and 800 °C. The red color may have been due to Fe_2_O_3_ present in the FA [62]. The GPM specimens’ surfaces became dark yellow, and concrete spalling occurred after 800 °C (see Figure 22f).

**Figure 23:**
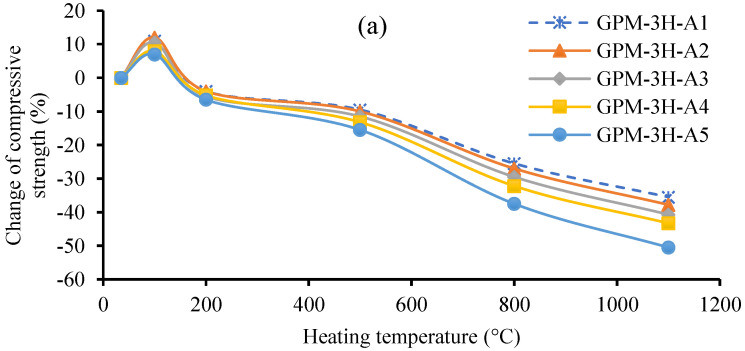

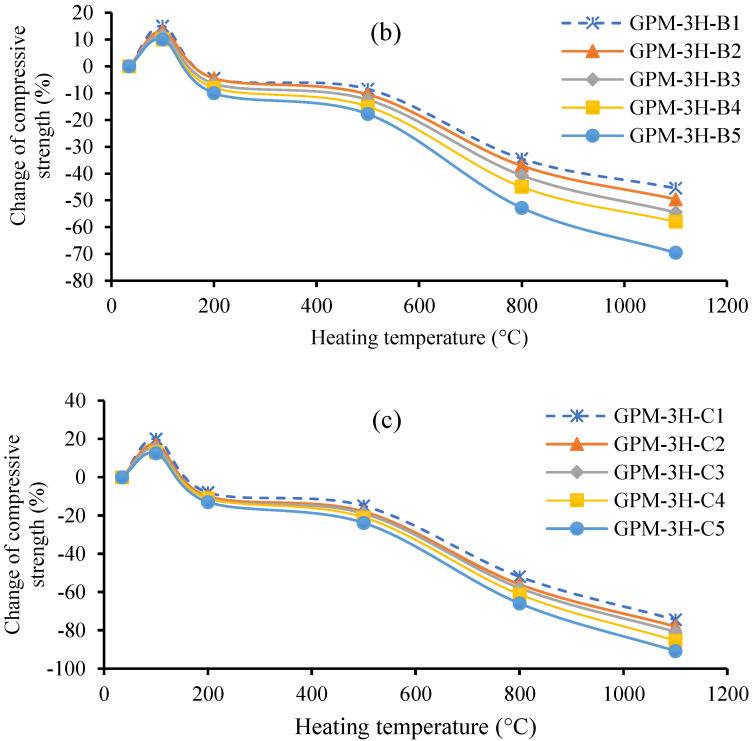
Change in compressive strength (%) vs. elevated temperature for the geopolymer material using various Na_2_SiO_3_-to-NaOH and FA-to-GGBS ratios with sand preheated at 110 °C: (**a**) Series A geopolymer paste; (**b**) Series B geopolymer paste; (**c**) Series C geopolymer paste.

Additionally, the construction material’s impact on the residual Cs after heating at elevated temperatures is also vital from a construction point of view. Figure 23a–c shows the change in Cs (%) vs. elevated temperature for the Series A, Series B, and Series C geopolymer material (see Table 4), respectively, using various SS-to-SH (%) and FA-to-GGBS ratios with sand preheated at 110 °C. Figure 23a–c shows that the Cs of the GPM increased after heating at 100 °C but decreased when the heating temperature was more than 100 °C in all GPM specimens as compared to the Cs at room temperature. Furthermore, it was also observed that the drop in the Cs (%) was more severe in the Series C GPM paste (Figure 23c) as compared to the Series B and Series A pastes. The Series A GPM exhibited less deterioration in the Cs as compared to the Series B and Series C GPM. It was also noted that after heating at 100 °C, the Cs of the Series C GPM paste increased from 12.5% to 20% with respect to initial values at room temperature. On the other hand, the Cs of the Series B and Series A GPM pastes were observed to drop from 15% to 10% and 11% to 7%, respectively. The Cs of the Series A, Series B, and Series C GPM pastes decreased from 25% to 37.5%, 34.5% to 52.8%, and 52% to 65.92%, respectively, after heating at 800 °C, and 35.5% to 50.5%, 45.5% to 69.6%, and 74.5% to 90.82%, respectively, after heating at 1100 °C as compared with initial values at room temperature. The Series C GPM paste contained a higher percentage of GGBS and was the weakest among the paste Series at elevated temperatures of more than 100 °C. Moreover, adding GGBS as a binder initially enhanced the Cs of the GPM at low temperatures but significantly reduced the Cs at elevated temperatures. This may have been due to the formation of C-S-H minerals in the GPM paste. Several researchers indicated that using GGBS as a binder significantly enhanced GPM’s rapid setting and strength development properties due to the production of C-S-H minerals [17,61]. Elevated temperature heating decomposed the C-S-H gel, and the loss of chemically bound water molecules, i.e., bonded with C-S-H minerals, in the form of evaporation led to the decrease in the residual Cs of the GPM [60].

Moreover, Figure 23a,b shows that the alkaline activator SS-to-SH ratio (%) did not cause any significant change in the residual Cs of any of the GPM pastes at the initial heating temperature. Nevertheless, at 800 °C and 1100 °C, the GPM paste had a higher SS-to-SH ratio (%), indicating more deterioration in the Cs as compared to the unexposed specimens in all GPM pastes. This may have been due to the spalling of the GPM at elevated temperatures (see Figure 22f). Spalling is the most complex characteristic of concrete, and it can occur at high temperatures [59]. Fletcher et al. [59] reported that spalling caused a steep deterioration in the mechanical strength of the concrete.

**Figure 24:**
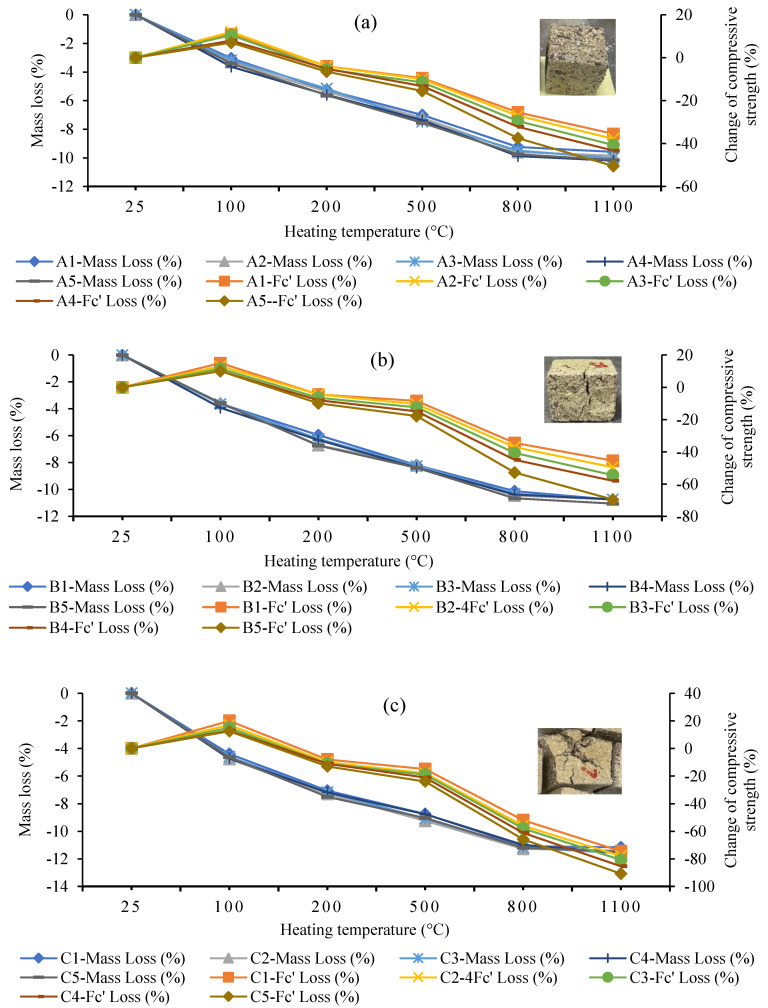
Relative change in mass loss (%) and compressive strength (%) of the geopolymer material using various Na_2_SiO_3_-to-NaOH and fly ash-to-GGBS ratios with sand preheated at 110 °C: (**a**) Series A geopolymer paste; (**b**) Series B geopolymer paste; (**c**) Series C geopolymer paste.

Figure 24 shows the relative change in mass loss (%) and compressive strength (%) of the GPM using various SS-to-SH (%) and FA-to-GGBS ratios with sand preheated at 110 °C: (a) Series A geopolymer paste; (b) Series B geopolymer paste; (c) Series C geopolymer paste. As can be observed in Figure 24, exposing the GPM to elevated temperatures decreased the Cs and led to mass loss in all GPM specimens. It can also be noted from Figure 24 that there was an almost linear downward trend in the Cs and mass loss in all GPM pastes due to heating. There was an exception: all GPM specimens exposed to 100 °C experienced an increase in the Cs, i.e., by 12%, 15%, and 20% in the Series A, Series B, and Series C GPM pastes, respectively, as compared to the unexposed GPM paste, although there was a reduction in mass in all GPM specimens. Figure 24c shows a sharp drop in the Cs at a temperature of 800 °C and 1100 °C, with only 34.08 and 9.18% of the Cs being retained, respectively, as compared to the unexposed specimens. The steep drop in the Series C GPM specimen followed the conversion of mass loss (%), and the change in Cs (%) was also observed in the Series C GPM after heating at 1100 °C. The residual Cs value observed without a considerable decrease in mass loss may result from high GPM paste spalling (Figure 24c). As a result, the bonding between the fine aggregate and geopolymer gel is weakened, promoting microcracks inside the GPM paste [59].

## 4. Conclusions

Phase 1 of the study examined how preheated sand affected the Cs of the geopolymer material (GPM), while phase 2 focused on monitoring how the different SS-to-SH (%) and FA-to-GGBS ratios affected the Cs of the GPM with preheated sand. The results indicate that preheated sand increased the Cs of the GPM compared to sand at room temperature (25 ± 2 °C) due to the heat energy increasing the reaction kinetics of the polymerization reaction with a similar curing period and FA-to-GGBS ratio. Preheated sand provided heat energy, which significantly accelerated the formation of polymers, resulting in a higher GPM Cs. Additionally, sand preheated at 110 °C produced better results than the other temperatures. Therefore, it may be concluded that sand preheated at 110 °C was optimal in terms of yielding a high GPM Cs compared to the other temperatures.

This research also addressed the effect of preheated sand and various SS-to-SH (%) and FA-to-GGBS ratios on the mechanical properties of the GPM. It was noted that increasing the amount of GGBS produces a more homogeneous, denser microstructure, which improved the Cs of the GPM. This may have been due to the different formations of calcium silicate hydrates (or C-S-H) in the GPM. A mixture of Na_2_SiO_3_ (SS) and NaOH (SH) produced better results than NaOH alone as an activator. When SS+SH was utilized as an activator, crystalline calcium silicate hydrates and an amorphous gel were produced. The presence of Na_2_SiO_3_ (water glass) enhanced the Cs of the GPM and the SS-to-SH (%) and FA-to-GGBS ratios of 5% and 1:3, respectively, by mass yielding the maximum Cs of 52.52 MPa in 3 h. It can also be concluded that the SS-to-SH ratio of 5% provided better results than the other SS-to-SH ratios in the form of Cs (see Figure 12). All of the results from phase 2 were confirmed in SEM and EDS micrograph analyses.

Temperatures up to 100 °C enhanced the Cs of the GPM compared to the GPM produced at room temperature. This increased strength was observed in all GPM specimens. A heating temperature of more than 100 °C decreased the Cs of the GPM, even to a level below the unexposed specimen. All specimens exhibited this trend. The maximum deterioration in Cs was noted to be 13.01%, 23.91%, 65.92%, and 98.82% after temperatures of 200 °C, 500 °C, 800 °C, and 1100 °C, respectively, in the Series C GPM compared to unexposed specimens. The mass loss (%) of the GPM paste at elevated temperatures followed a similar pattern. The conversion of mass loss (%) and the change in Cs (%) were also observed in the Series C GPM at 1100 °C. Therefore, replacing FA with GGBS by up to 75% enhanced the rapid Cs characteristics but reduced the thermal stability of the GPM paste at elevated temperatures.

The SS-to-SH ratio (%) did not have any impact on the mass loss of the GPM paste exposed to elevated temperature in all specimens. Furthermore, it was also observed that the SS-to-SH ratio (%) did not have any material effect on the Cs of the GPM paste exposed to lower elevated temperatures of up to 500 °C in all samples. A significant loss of Cs was noted after temperatures of 800 °C and 1100 °C due to GPM spalling, and raising the SS-to-SH ratio (%) in the GPM matrix enhanced the Cs loss at elevated heating temperatures.

In addition, the GPM with FA-to-GGBS and SS-to-SH ratios of 1:3 and 5%, respectively, is the best mixture for the restoration of bridge deck expansion joints. It is a green concrete, it reaches a compressive strength of 52.52 MPa in 3 h, it has a flow rate of 30.5%, and industrial waste is one of its main constituents. Moreover, considering fire loading, a GPM with FA-to-GGBS and SS-to-SH ratios of 1:2 and 5%, respectively, is the best matrix for the construction of small building elements. This study may be valuable for practical engineering to solve the rehabilitation problem of bridge deck expansion joints in a short time, and emergency repair work during natural disasters. However, as a result of the limited flow rate and very short setting time, the developed GPM material may not be suitable for large constructions. Therefore, it is necessary to study rapid-strength green concrete compositions further.

## Figures and Tables

**Table 1:** Specification of liquid sodium silicate (Na_2_SiO_3_).

Specification of Liquid Sodium Silicate (Na_2_SiO_3_),	Quantity (%)
Specific Weight	1.526 g/cm^3^
SiO_2_%	28–30
Na_2_O%	9–10
Fe%	Under 0.02
pH value	12.00

**Table 2:** Composition of class-F fly ash (FA) and S-4000 GGBFS as determined by XRD (mass %).

Chemical Composition (% by Mass)	SiO_2_	Al_2_O_3_	Fe_2_O_3_	SO_3_	CaO	As	S	Pb	Loss on Ignition
Class-F fly ash (FA)	58.58	25.47	2.37	0.96	0.00	6.36	0.96	5.63	0.63
S-4000 GGBS	21.64	8.53	0.81	0.91	68.11	-	-	-	0.11

**Table 3:** Material properties of fly ash (FA), GGBS, and natural river sand.

Material Type	Property	Results
Class-F fly ash	Specific gravity (ASTM C618-16)	1.45
S-4000 ground granulated blast furnace slag (GGBS)	Specific gravity (ASTM C618-16)	2.59
Natural river sand	Specific gravity (ASTM C128-15)	2.63 (SSD) 2.76 (OD)
Natural river sand	Water absorption (%) (ASTM C128-15)	2.88
Natural river sand	Fineness modulus (ASTM C33/C33M-13)	2.63

Note: SSD: saturated surface-dry; OD: oven dry.

**Table 4:** Experimental geopolymer material mix proportion by weight.

	Specimen ID	FA-to-GGBS Ratio (Mass)	FA (g)	GGBS (g)	SS Solution (g)	SH-10M (g)	SS-to-SH Ratio	Preheated Sand	C_t_ (Oven)	C_p_ (h)	Al-to-Bi Ratio (Mass)	Li-to-S Ratio (Mass)	Bi-to-S Ratio (Mass)	MR SiO_2_ to Al_2_O_3_	MR CaO to SiO_2_
Phase 1	10M-GPM-0	1:1	185.6	185.6	-	269.67	-	(25 ± *2* °C)	50 °C	1, 2, 3, 24	0.726	0.30	0.42	2.36	0.85
	10M-GPM-50	1:1	185.6	185.6	-	269.67	-	50 °C	50 °C	“	0.726	0..30	0.42	2.36	0.85
	10M-GPM-65	1:1	185.6	185.6	-	269.67	-	65 °C	50 °C	“	0.726	0.30	0.42	2.36	0.85
	10M-GPM-80	1:1	185.6	185.6	-	269.67	-	80 °C	50 °C	“	0.726	0.30	0.42	2.36	0.85
	10M-GPM-100	1:1	185.6	185.6	-	269.67	-	100 °C	50 °C	“	0.726	0.30	0.42	2.36	0.85
	10M-GPM-110	1:1	185.6	185.6	-	269.67	-	110 °C	50 °C	“	0.726	0.30	0.42	2.36	0.85
	10M-GPM-120	1:1	185.6	185.6	-	269.67	-	120 °C	50 °C	“	0.726	0.30	0.42	2.36	0.85
	10M-GPM-135	1:1	185.6	185.6	-	269.67	-	135 °C	50 °C	“	0.726	0.30	0.42	2.36	0.85
				Series A											
Phase 2	GPM-3H-A1	1:1	185.6	185.6	0	269.67	0%	110 °C	50 °C	3 h	0.726	0.30	0.42	2.36	0.85
	GPM-3H-A2	1:1	185.6	185.6	13.4	256.27	5%	110 °C	50 °C	“	0.726	0.30	0.42	2.41	0.83
	GPM-3H-A3	1:1	185.6	185.6	26.96	242.71	10%	110 °C	50 °C	“	0.726	0.30	0.42	2.46	0.81
	GPM-3H-A4	1:1	185.6	185.6	53.93	215.74	20%	110 °C	50 °C	“	0.726	0.30	0.42	2.57	0.78
	GPM-3H-A5	1:1	185.6	185.6	80.89	188.78	30%	110 °C	50 °C	“	0.726	0.30	0.42	2.67	0.75
					Series B										
Phase 2	GPM-3H-B1	1:2	123.73	247.46	0	269.67	0%	110 °C	50 °C	“	0.726	0.30	0.42	2.4	1.34
	GPM-3H-B2	1:2	123.73	247.46	13.4	256.27	5%	110 °C	50 °C	“	0.726	0.30	0.42	2.46	1.30
	GPM-3H-B3	1:2	123.73	247.46	26.96	242.71	10%	110 °C	50 °C	“	0.726	0.30	0.42	2.52	1.27
	GPM-3H-B4	1:2	123.73	247.46	53.93	215.74	20%	110 °C	50 °C	“	0.726	0.30	0.42	2.64	1.21
	GPM-3H-B5	1:2	123.73	247.46	80.89	188.78	30%	110 °C	50 °C	“	0.726	0.30	0.42	2.77	1.16
					Series C										
Phase 2	GPM-3H-C1	1:3	92.8	278.4	0	269.67	0%	110 °C	50 °C	“	0.726	0.30	0.42	2.42	1.65
	GPM-3H-C2	1:3	92.8	278.4	13.4	256.27	5%	110 °C	50 °C	“	0.726	0.30	0.42	2.49	1.61
	GPM-3H-C3	1:3	92.8	278.4	26.96	242.71	10%	110 °C	50 °C	“	0.726	0.30	0.42	2.56	1.57
	GPM-3H-C4	1:3	92.8	278.4	53.93	215.74	20%	110 °C	50 °C	“	0.726	0.30	0.42	2.69	1.49
	GPM-3H-C5	1:3	92.8	278.4	80.89	188.78	30%	110 °C	50 °C	“	0.726	0.30	0.42	2.83	1.41

Note: FL: fly ash; GGBS: ground granulated blast-furnace Slag; SS: sodium silicate; SH: sodium hydroxide; C_t_: curing temperature; C_p_: curing period; h: hours; Al: alkaline liquid; Bi: binder; Li: liquid; S: solid; MR: molar ratio.

**Table 5:** Compressive strength test results of the geopolymer material (GPM) using room-temperature sand and preheated sand after 1 h, 2 h, 3 h, and 1 d of hot air curing at a constant temperature of 50 °C.

Specimen ID	Flow (mm)	Compressive Strength (MPa)			
		1 h	2 h	3 h	24 h
10M-GPM-0	182.00	3.06	12.83	19.82	24.99
10M-GPM-50	180.00	4.71	18.07	28.35	35.18
10M-GPM-65	178.00	7.30	22.21	31.00	40.57
10M-GPM-80	175.00	7.89	22.84	31.92	44.50
10M-GPM-100	171.00	14.69	26.13	33.71	45.60
10M-GPM-110	168.0	17.23	29.19	42.31	51.36
10M-GPM-120	165.00	13.58	27.86	36.90	48.18
10M-GPM-135	161.50	8.08	21.03	29.19	37.92

**Table 6:** Setting time, flow rate, compressive strength, standard deviation (SD), and the coefficient of variation (CoV) of the GPM using various Na_2_SiO_3_-to-NaOH and FA-to-GGBS ratios with sand preheated at 110 °C and cured in a hot oven at 50 °C.

Sr. No	Mix ID	FA-to-GGBS (by Mass)	Na_2_SiO_3_-to-NaOH (SS-to-SH) (by Mass)	Initial Setting Time (min)	Final Setting Time (min)	Flow Rate (%)	SiO_2_ to Al_2_O_3_	CaO to SiO_2_	f’c (Mean) in 3 h (MPa)	SD	CoV (%)
		Series A									
1	GPM-3H-A1	(1:1)	0%	17	59	37.0	2.36	0.85	42.31	1.3	3.07
2	GPM-3H-A2	(1:1)	5%	16.5	54	36.5	2.41	0.83	45.61	1.07	2.35
3	GPM-3H-A3	(1:1)	10%	15	48	34.5	2.46	0.81	44.14	0.266	0.59
4	GPM-3H-A4	(1:1)	20%	13.5	43	33.0	2.57	0.78	37.88	1.21	3.21
5	GPM-3H-A5	(1:1)	30%	11.5	37	30.5	2.67	0.75	32.04	1.97	6.15
		Series B									
1	GPM-3H-B1	(1:2)	0%	14	45	34.0	2.4	1.34	44.59	2.05	4.60
2	GPM-3H-B2	(1:2)	5%	13.5	39	33.0	2.46	1.30	48.61	0.71	1.45
3	GPM-3H-B3	(1:2)	10%	12	36	32.0	2.52	1.27	45.89	1.51	3.29
4	GPM-3H-B4	(1:2)	20%	10.5	31	30.0	2.64	1.21	41.02	1.45	3.54
5	GPM-3H-B5	(1:2)	30%	9.0	28	27.5	2.77	1.16	38.12	1.28	3.37
		Series C									
1	GPM-3H-C1	(1:3)	0%	13.0	35	31.0	2.42	1.65	45.95	0.99	2.14
2	GPM-3H-C2	(1:3)	5%	12.5	31	30.5	2.49	1.61	52.52	1.92	3.65
3	GPM-3H-C3	(1:3)	10%	10.5	27	28.0	2.56	1.57	46.70	5.10	10.92
4	GPM-3H-C4	(1:3)	20%	9.0	23	29.0	2.69	1.49	40.6	1.28	3.14
5	GPM-3H-C5	(1:3)	30%	7.5	19.5	25.5	2.83	1.41	35.55	1.41	3.96

## Data Availability

All data is available on genuine request.
